# Add-On Therapy with Ertapenem in Infections with Multidrug Resistant Gram-Negative Bacteria: Pediatric Experience

**DOI:** 10.1155/2017/8096420

**Published:** 2017-07-26

**Authors:** Sevgen Tanır Basaranoglu, Yasemin Ozsurekci, Kubra Aykac, Kamile Oktay Arıkan, Ayse Buyukcam, Ali Bulent Cengiz, Mehmet Ceyhan, Ates Kara

**Affiliations:** Pediatric Infectious Disease Department, İhsan Doğramacı Children's Hospital, Hacettepe University, Ankara, Turkey

## Abstract

Optimal therapy for infections with carbapenem resistant GNB is not well established due to the weakness of data. Patients presenting with bloodstream infections caused by multidrug resistant* Klebsiella pneumoniae* were treated with a combination treatment. Optimal therapy for infections with carbapenem resistant Gram-negative bacteria is a serious problem in pediatric patients. We presented three cases who were successfully treated with addition of ertapenem to the combination treatment for bacteremia with multidrug resistant* Klebsiella pneumoniae*. Dual carbapenem treatment approach is a new approach for these infections and requires more data in children.

## 1. Introduction

The emergence of multidrug resistant (MDR) Gram-negative bacteria (GNB) poses a significant threat for global public health because of the limited therapeutic options for treatment. Optimal therapy for infections with carbapenem resistant GNB is not well established [1], while increasing amount of evidence suggests that combination therapy is more effective than monotherapy. In addition, promising novel agents are under development for MDR-GNB, such as ceftazidime-avibactam. This paper presents three pediatric cases of MDR-GNB infection successfully treated with dual carbapenem for bacteremia caused by MDR-GNB.

## 2. Case  1

**Figure 1:**
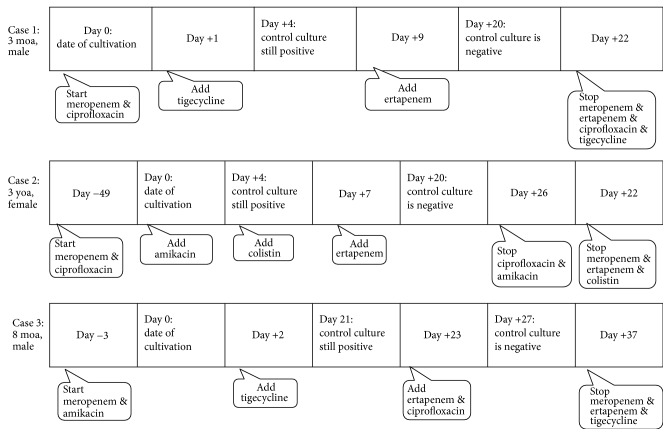
Timeline of treatment for Gram-negative bacteria and follow-up.* Dosages of Drugs.* Meropenem: 20–40 mg/kg every 8 hours; ciprofloxacin: 6–8 mg/kg every 8 hours; tigecycline: 1–1.5 mg/kg every 12 hours; ertapenem: 15 mg/kg every 12 hours; amikacin: 15 mg/kg every 24 hours; colistin: 2.5–5 mg/kg/day in 3 divided doses.

A three-month-old male was admitted with chronic diarrhea and a diagnosis of infantile hemochromatosis. He was hospitalized for investigational studies for the underlying condition. In the follow-up, he exhibited fever, as well as signs and symptoms of sepsis. He had to be transferred to the pediatric intensive care unit (PICU). A blood culture performed at the beginning of the fever revealed MDR* Klebsiella pneumoniae *(*K. pneumoniae*) (Table 1). Because the bacteria displayed a multidrug resistant profile, meropenem and ciprofloxacin were commenced (Figure 1). On the following day, tigecycline was added. On the fourth day of treatment, when the control blood culture was still positive for* K. pneumoniae*, ertapenem was added to the combination. The blood culture was negative on the 11th day of ertapenem treatment, and the patient clinically improved. All treatment was ceased on the 22nd day of ertapenem treatment (Figure 1).

## 3. Case  2

The second case was a three-year-old female patient who had been followed up in the PICU after corrective cardiac surgery for tetralogy of Fallot. She was on mechanical ventilation with a central venous catheter. She had been on meropenem, ciprofloxacin, and vancomycin treatment for systemic inflammatory response syndrome postoperatively when MDR* K. pneumoniae* resulted in both peripheral and catheter-driven blood cultures (Table 1). Her central venous catheter was removed, and a new one was placed into another location. Since the microorganism displayed colistin susceptibility, colistin was added on the fourth day due to ongoing positive blood culture (Figure 1). On the seventh day, the patient was still bacteremic; therefore, ertapenem was commenced in addition to meropenem, ciprofloxacin, amikacin, and colistin. A control blood culture was negative on the fourth day of the dual carbapenem regimen. Complete recovery from infection was achieved on the 25th day of ertapenem treatment.

## 4. Case  3

An eight-month-old male was diagnosed with primary hemophagocytic lymphohistiocytosis, and he had undergone bone marrow transplantation after chemotherapeutics. In the first month of transplantation, while he was waiting for bone marrow recovery and was on the third day of meropenem and amikacin treatments because of the suspicion of catheter-associated bloodstream infection (CRBSI), he developed a fever. MDR* K. pneumoniae* was isolated both from a culture of peripheral blood and from a culture of blood from the central venous catheter (Table 1). His central venous catheter was removed, and a new one was placed into another central vein. Although tigecycline was added based on the patient's susceptibility profile in light of the CRBSI, blood culture was still positive for the microorganism on the 21st day. Ertapenem was started on the 23rd day of cultivation. On the fourth day of dual treatment, a negative blood culture was achieved. Treatment against GNB was successfully stopped on the 37th day (Figure 1).

## 5. Discussion

To the best of our knowledge, these are the first reported pediatric cases with bacteremia caused by carbapenemase-producing* K. pneumoniae* and successfully treated with a double-carbapenem regimen. GNBs are becoming increasingly nonsusceptible to carbapenems, due to the acquisition of new resistance mechanisms, usually accompanied by resistance to all *β*-lactam agents and often to many other classes, such as quinolones and aminoglycosides. Furthermore, treatment options for these microorganisms are extremely limited, and no consensus has been established for their management [1, 2]. In the context of infections with these microorganisms—especially those resistant to carbapenems—the role of double-carbapenem regimens containing ertapenem has been previously reviewed [3–5]. Ertapenem is used as an indicator of the expression of carbapenemases, resulting from the ease of hydrolysis compared to other members of the group. Because carbapenemases have an increased affinity for ertapenem, it is believed to be the least active agent against carbapenemase-producing* K. pneumoniae*, and it behaves like a suicide substrate [6–8]. This belief is the rationale for the use of combination carbapenem therapy.

The mainstay of therapy for patients with bacteremia remains antimicrobial therapy, together with optimal management of the consequences, including treatment of shock and surgical treatment of infection sites, such as drainage of abscesses and removal of intravascular devices. In our cases, in addition to arrangement of combinations of antimicrobial agents, replacement of central venous catheters into other veins may have contributed to the successful results.

Our institution cares for clinically high-risk patient groups, including those suffering from primary immunodeficiencies, hematologic/oncologic malignancies, and many chronic conditions requiring long hospital stays. The possibility of encountering MDR-GNB in these patients is ever-increasing, and the situation necessitates new therapy options. For the present pediatric cases, a combination of ertapenem with meropenem, despite individual resistance to both, was a successful choice. However, larger studies are required in order to lighten the consequences of this therapy.

## Figures and Tables

**Table 1:** Demographic characteristics of patients and features of resistant microorganisms.

	Age	Underlying diagnosis	Clinical syndrome	Site of cultivation	Microorganism	Antibiotic sensitivity profile				
						MPM/MIC	ERT/MIC	AMK/MIC	CPR/MIC	TGC/MIC
Case 1	3 moa	Infantile hemochromatosis	Bacteremia	Blood culture	*Klebsiella pneumoniae*	R/>6	R/>8	IR/16	R/>2	S/1
Case 2	3 yoa	Operated tetralogy of Fallot	Bacteremia	Blood culture	*Klebsiella pneumoniae*	R/>32	R/>32	R/>64	R/>4	R/>2
Case 3	8 moa	Primary hemophagocytic syndrome (after bone marrow transplantation)	Bacteremia	Blood culture	*Klebsiella pneumoniae*	R/>32	R/>32	R/>64	R/>4	S/2

MPM: meropenem; ERT: ertapenem; AMK: amikacin; TGC: tigecycline; CPR: ciprofloxacin; R: resistant; S: sensitive; IR: intermediate resistance; MIC: minimum inhibitory concentration (mg/L); yoa: years of age; moa: months of age.
